# Serine/Threonine Kinase 17A Is a Novel Candidate for Therapeutic Targeting in Glioblastoma

**DOI:** 10.1371/journal.pone.0081803

**Published:** 2013-11-28

**Authors:** Pingping Mao, Mary P. Hever-Jardine, Gilbert J. Rahme, Eric Yang, Janice Tam, Anita Kodali, Bijesh Biswal, Camilo E. Fadul, Arti Gaur, Mark A. Israel, Michael J. Spinella

**Affiliations:** 1 Department of Pharmacology and Toxicology, Geisel School of Medicine at Dartmouth, Hanover, New Hampshire, United States of America; 2 Genetics, Geisel School of Medicine at Dartmouth, Hanover, New Hampshire, United States of America; 3 Neurology, Geisel School of Medicine at Dartmouth, Hanover, New Hampshire, United States of America; 4 Pediatrics, Geisel School of Medicine at Dartmouth, Hanover, New Hampshire, United States of America; 5 Norris Cotton Cancer Center, Lebanon, New Hampshire, United States of America; University of Portsmouth, School of Pharmacy & Biomedical Sciences, United Kingdom

## Abstract

STK17A is a relatively uncharacterized member of the death-associated protein family of serine/threonine kinases which have previously been associated with cell death and apoptosis. Our prior work established that STK17A is a novel p53 target gene that is induced by a variety of DNA damaging agents in a p53-dependent manner. In this study we have uncovered an additional, unanticipated role for STK17A as a candidate promoter of cell proliferation and survival in glioblastoma (GBM). Unexpectedly, it was found that STK17A is highly overexpressed in a grade-dependent manner in gliomas compared to normal brain and other cancer cell types with the highest level of expression in GBM. Knockdown of STK17A in GBM cells results in a dramatic alteration in cell shape that is associated with decreased proliferation, clonogenicity, migration, invasion and anchorage independent colony formation. STK17A knockdown also sensitizes GBM cells to genotoxic stress. STK17A overexpression is associated with a significant survival disadvantage among patients with glioma which is independent of age, molecular phenotype, IDH1 mutation, PTEN loss, and alterations in the p53 pathway and partially independent of grade. In summary, we demonstrate that STK17A provides a proliferative and survival advantage to GBM cells and is a potential target to be exploited therapeutically in patients with glioma.

## Introduction

Gliomas are the most frequent primary brain tumors and include a variety of histological types based on morphological criteria and are graded on a scale of I-IV [1]. The most common and lethal gliomas are grade IV astrocytoma called glioblastoma (GBM). The median survival of patients with GBM treated with the standard surgery, radiation and temozolomide is 12-15 months [2-5]. Clearly more effective therapies are needed and identification of new molecular targets is one key strategy to achieve this goal.

Gliomas exhibit frequent distinct alterations in genetic pathways including those of p53, NF1, PTEN, IDH1, EGFR and PDFGR [6,7]. The p53 pathway is frequently compromised in gliomas due to mutations in p53, deletion of CDKN2A, or amplification of MDM2/MDM4 [8]. GBM is also classified into 4 different subtypes (classical, mesenchymal, proneural and neural) based on gene expression profiling [9]. Drugs that target known genetic abnormalities in glioma and GBM have failed to show significant clinical utility [10]. For example, the EGFR receptor resides on chromosome 7 and is commonly amplified in GBM yet clinical benefits of EGFR inhibitors have been limited [11]. Uncovering new and previously unrecognized molecular targets altered in GBM may provide opportunities to improve the outcome of patients with malignant high-grade gliomas. 

STK17A is a member of the death associated protein (DAP) family of serine/threonine protein kinases which includes the prototypic family member DAPK1, along with DAPK2 and DAPK3 (ZIPK) [12-14]. STK17A (DRAK1) and STK17B (DRAK2) are more distantly related and less characterized members of the DAPK family [12-14]. DAPK1 is a multi-domain cytosolic actin filament-associated calcium/calmodulin-dependent, serine/threonine kinase that has a known regulatory role in cytoskeletal dynamics, apoptosis, autophagy, cell adhesion and motility [15-17]. DAPK1 is also a proposed tumor suppressor gene [16]. Other than the regulatory light chain of myosin II (MLC), the key *in vivo* substrates of DAPK1 are unclear [12]. In contrast to DAPK1, very little is known about the biological role of STK17A. Although possessing moderate homology in the catalytic kinase domain, STK17A lacks many of the regulatory domains of DAPK1 including the death domain and the Ca^2+^/calmodulin regulatory domain [12-14]. We have previously shown that STK17A is a direct p53 target gene that plays a role in cisplatin-mediated toxicity of testicular cancer cells in association with regulation of reactive oxygen species (ROS) [18]. 

Here we provide evidence for an unanticipated oncogenic role for STK17A in glioma. STK17A was highly expressed in a grade-dependent manner in gliomas when compared to normal brain. Interestingly, knockdown of STK17A in GBM cells results in a dramatic alteration in cell shape that is associated with decreased proliferation, clonogenicity, migration, invasion and anchorage independent colony formation. Importantly, STK17A knockdown also sensitized GBM cells to genotoxic stress and STK17A overexpression was associated with a significant survival disadvantage among patients with glioma. These data support a role for STK17A as a new, previously unrecognized kinase target in GBM. 

## Results

### STK17A is highly expressed in GBM and human GBM cell lines

**Figure 1:**
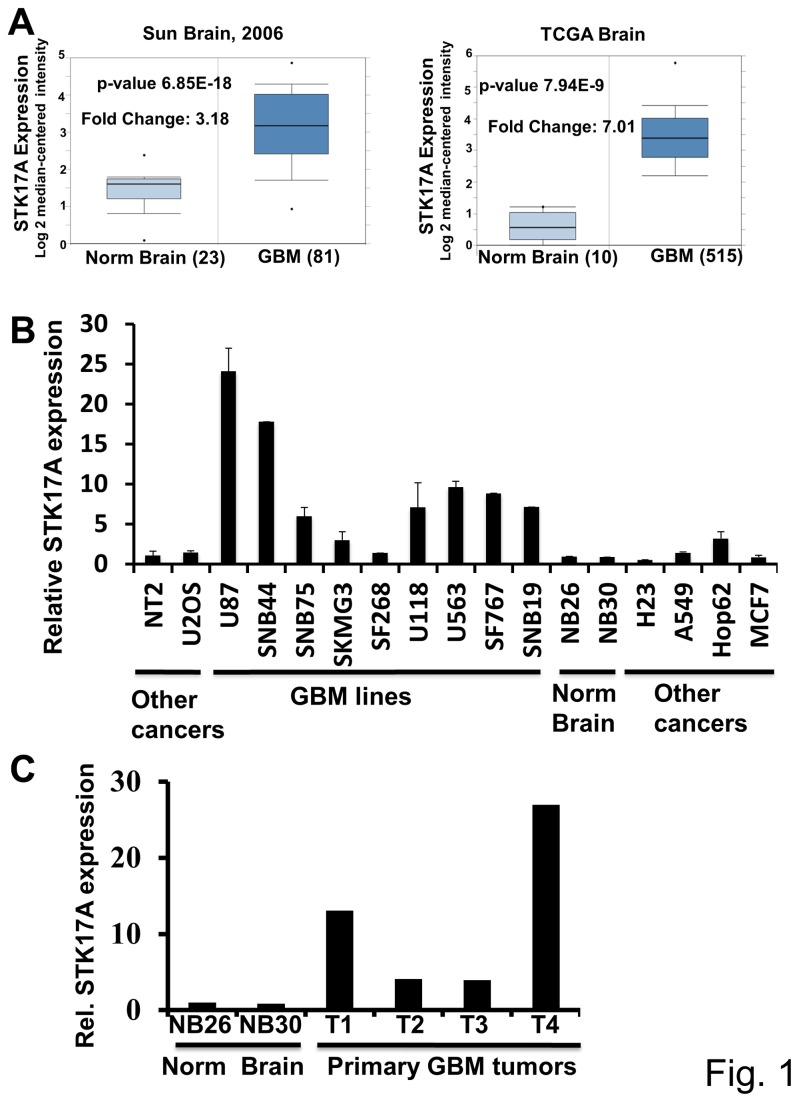
STK17A is overexpressed in GBM. **A**, Data from Sun Brain [19], and TCGA Brain (https://tcga-data.nci.nih.gov/tcga/tcgaHome2.jsp) comparing microarray-based STK17A expression from clinical nonmalignant brain specimens (Norm. Brain) and clinical GBM indicating high expression of STK17A in GBM. **B**, Real-time PCR analysis of STK17A expression in GBM cell lines compared to expression in other cancer types and normal brain. Expression is normalized to GAPDH. Bars are the average of triplicate or duplicate biological replicates except for normal brain RNA which is the average of technical duplicates. Error bars are SD. All cell lines are human. Non-GBM cell lines are as follows: NT2 (NT2/D1), embryonal carcinoma; U2OS, osteosarcoma; H23, A549, and Hop62, lung adenocarcinomas; MCF7, breast cancer. **C**, Real-time PCR analysis of STK17A expression in normal brain mRNA and four clinical GBM samples from Dartmouth Hitchcock Medical Center. Expression is normalized to GAPDH. The bars represent the averages of technical duplicate determinations.

**Figure 2:**
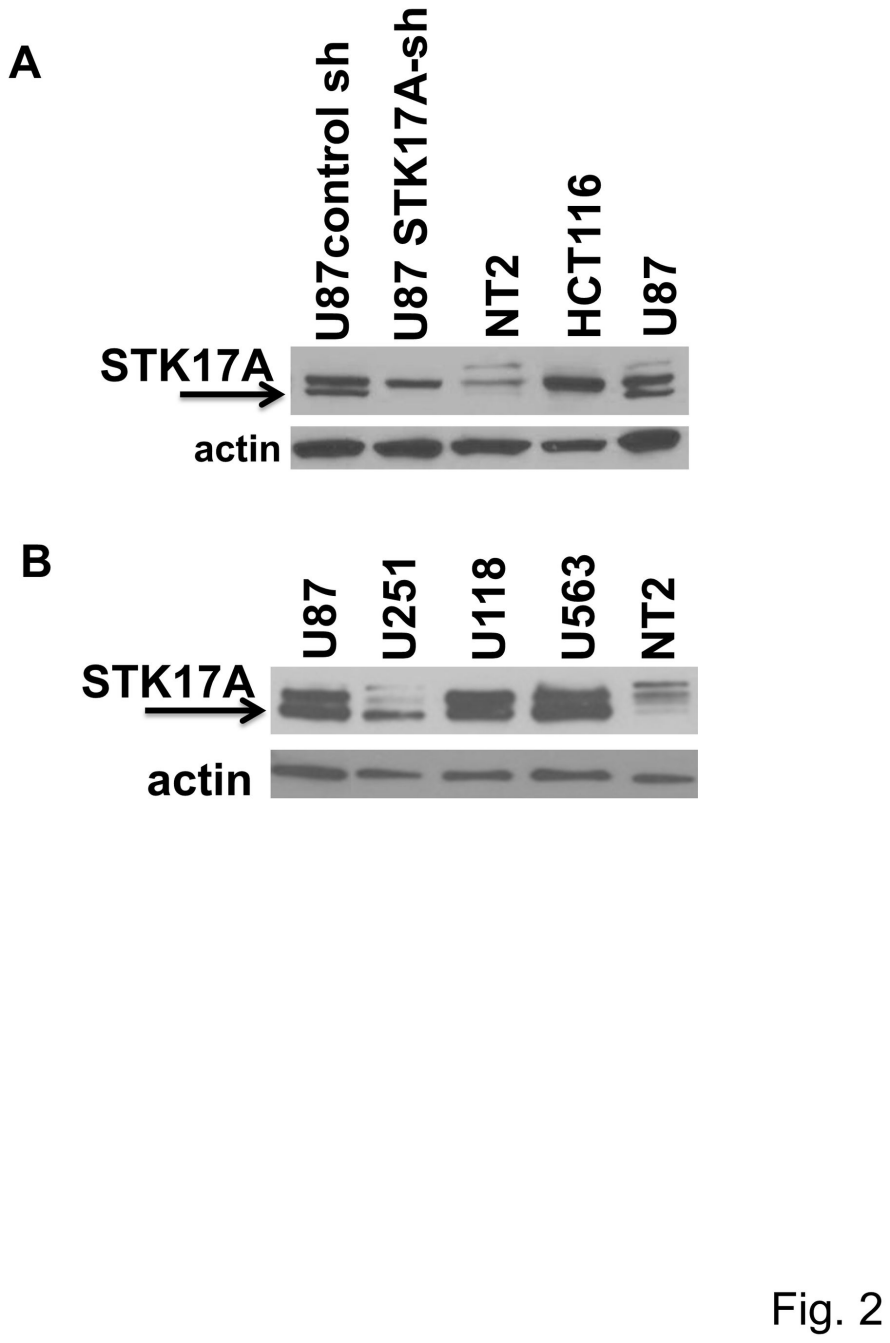
STK17A protein is overexpressed in GBM cells lines compared to NT2/D1 and HCT116 cells. **A**, **B**, Western analysis comparing STK17A expression in U87, U251, U118 and U563 GBM cells compared to NT2/D1 human embryonal carcinoma and HCT116 colon cancer cells. Arrow indicates STK17A specific band identified by the diminished signal in U87 cells stably expressing STK17A shRNA.

**Figure 3:**
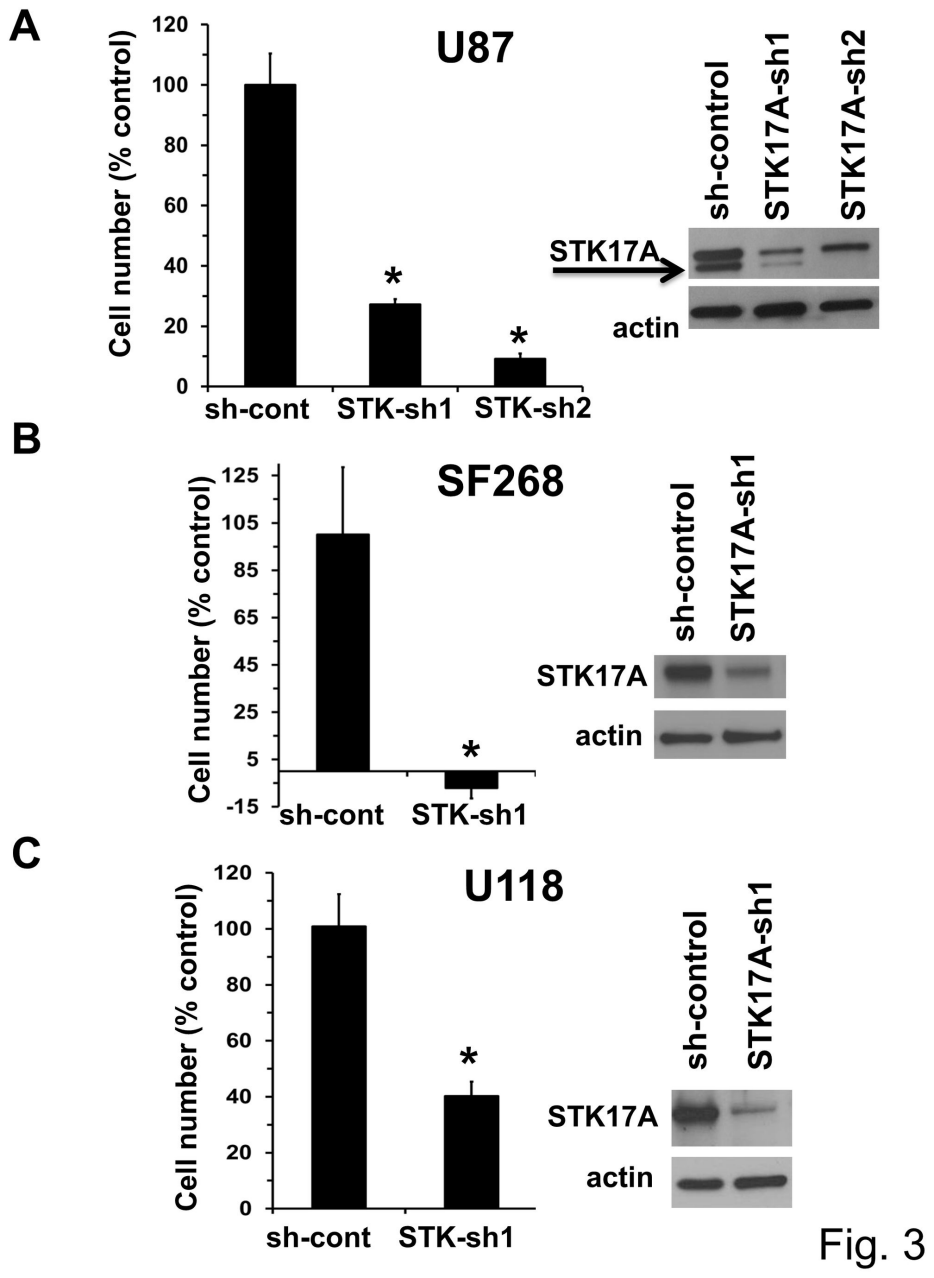
STK17A knockdown results in decreased GBM cell proliferation. U87, SF268 and U118 GBM cells stably expressing STK17A shRNA or a control shRNA were assessed for changes in cell proliferation by cell counting 4.5 days after plating. Cell counts were normalized to cell counts after 0.5 days of plating to control for plating errors and differences in cell adherence. Bars are the average of three biological replicates and error bars are SD. *, p < 0.05. Data are representative of at least two independent experiments. To the right of each graph the extent of STK17A knockdown was assessed by Western analysis.

**Figure 4:**
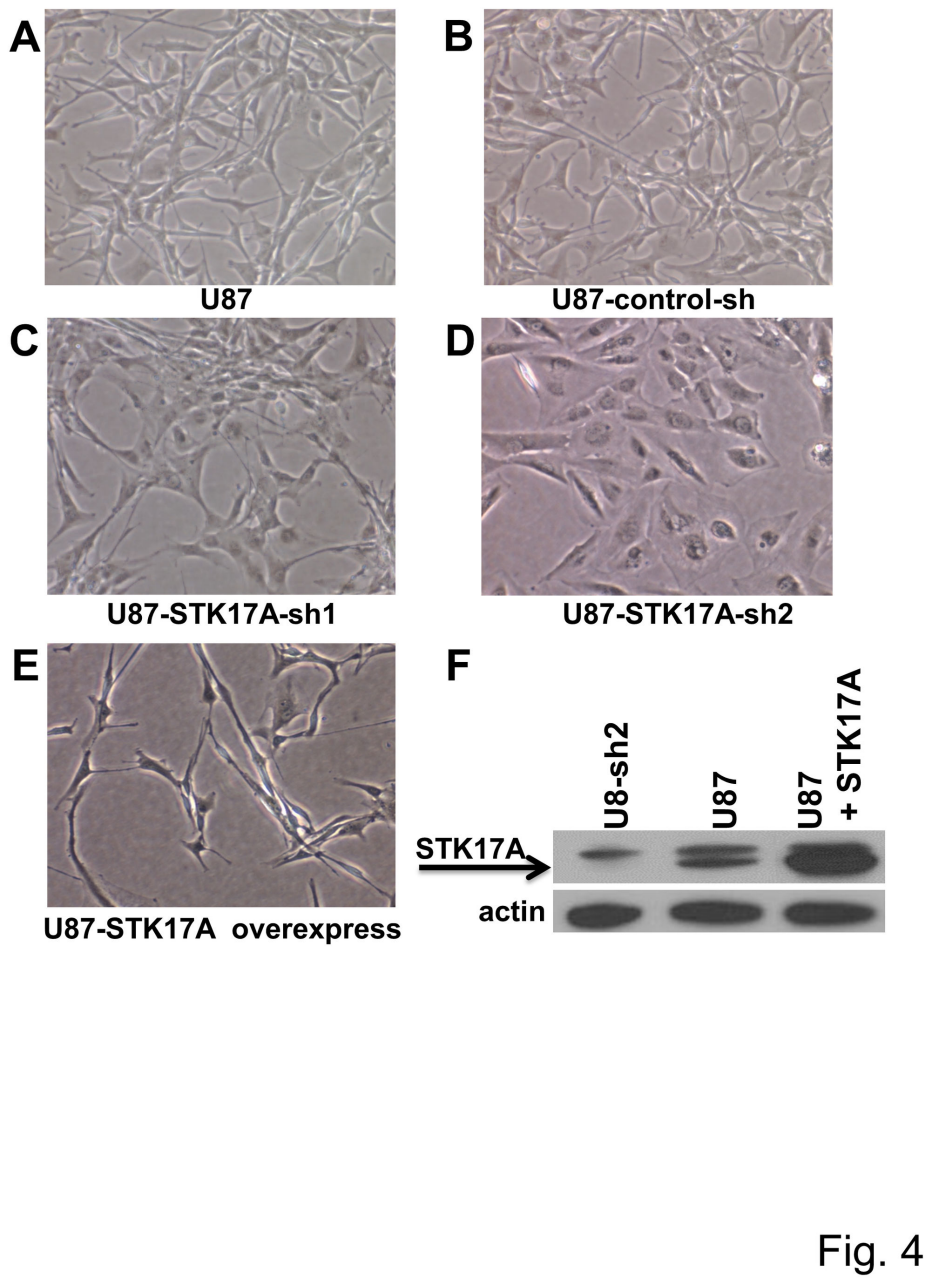
Knockdown or overexpression of STK17A alters cell morphology of U87 cells. Representative micrographs of parental U87 cells (**A**), U87 cells stably expressing control shRNA (**B**), two distinct STK17A shRNAs (**C**, **D**), or U87 cells stably overexpressing STK17A (**E**). Cells become large, flatter and more rounded upon STK17A knockdown while cells take on an elongated, needle-shaped appearance upon STK17A overexpression. Representative of three independent derivations of each cell line. Pictures were taken at 10X magnification on a NIKON ELWD microscope. **F**, Western analysis of STK17A knockdown and overexpression. Efficiency of stable knockdown is depicted in Figure 3.

Based on our prior interest in STK17A as a novel p53 target gene, *in silico* analysis was performed to determine whether STK17A expression was deregulated in human cancers. Unexpectedly, the most significant alteration found was prominent overexpression of STK17A in human glioma. In the Oncomine database 5 out of 7 studies documented high STK17A expression in GBM compared to normal brain (2.9-fold to 7.0-fold increase) (Figure 1A and Figure S1) [19-22]. An additional study indicated a 4.8-fold increase in STK17A expression in anaplastic oligodendroglioma compared to normal brain (Figure S1) [23]. Further, the Cancer Cell Line Encyclopedia (CCLE) database demonstrated that of 1062 cell lines representing 37 distinct cancer types, glioma cell lines express the highest levels of STK17A (Figure S1) [24]. These findings based on microarray gene expression data were confirmed by directly assessing STK17A mRNA and protein expression which demonstrated substantial overexpression (up to 20-fold) of STK17A in GBM cell lines compared to non-GBM cancer cell lines originating from other organs and normal brain (Figure 1B, Figure 2 and Figure S2). Note that some but not all cells lines demonstrate two prominent bands in Western analysis. The lower band was validated as representing STK17A based on its sensitivity to shRNA inhibition and our finding that overexpression of STK17A increases the intensity of this lower band (Figure 2, Figure 3 and Figure 4). We also confirmed that STK17A mRNA levels were increased in GBM tumor tissue compared to STK17A levels in normal brain (Figure 1C). 

### STK17A knockdown in GBM cells results in decreased cell proliferation, morphological changes, actin stress fiber formation, and inhibition of cell motility/invasion

**Figure 5:**
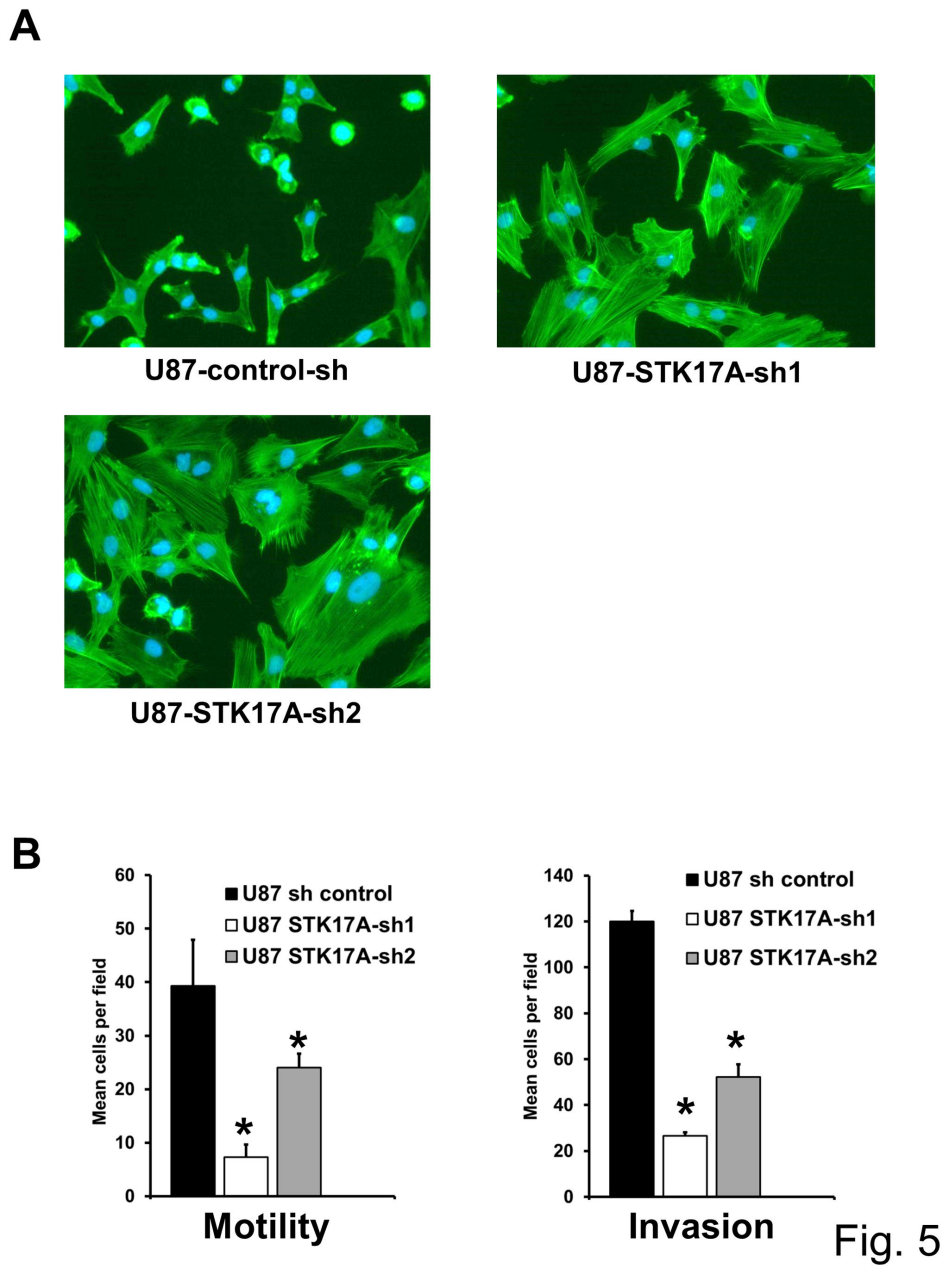
STK17A knockdown results in the formation of actin stress fibers and inhibition of cell motility and invasion. **A**, Representative fluorescent images of U87 cells stably expressing control shRNA and two distinct STK17A shRNAs. Actin is stained green and nuclei are stained blue. Note the larger size of STK17A knockdown cells and the presence of actin stress fibers. Extent of knockdown is depicted in Figure 3. This morphologic phenotype was apparent in independent derivations of the cell lines. Pictures were taken at 20X magnification on a NIKON ELWD fluorescent microscope. **B**, STK17A knockdown decreases cell migration and invasion of U87 cells. Cell migration and invasion was assessed as described in Methods. Bars are the average of biological triplicates and error bars are SD. *, p < 0.05. Representative of two independent experiments.

To assess the importance of STK17A in GBM cell proliferation, GBM cells with stable shRNA knockdown of STK17A were generated. In U87, SF268, U118, A172, and U563 GBM lines knockdown of STK17A results in substantial decreased cell proliferation (Figure 3 and Figure S3). The decreased proliferation was associated with a consistent change in morphology such that cells became less spindle shaped and assumed a larger, more rounded and flattened appearance (Figure 4 and Figure S4). Interestingly overexpression of STK17A in GBM cells resulted in an exaggerated spindle-like phenotype as the cells became elongated, needle shaped and appeared more three-dimensional than wild-type cells (Figure 4E, Figure S4). The phenotype of STK17A knockdown and overexpressing GBM cells is consistent with the known role of the Drosophila ortholog of STK17A, DRAK, in regulating cell shape, actin/myosin dynamics and cytoskeletal changes [25-27]. Interestingly, knockdown of STK17A in GBM cells resulted in increased formation of actin stress fibers (Figure 5A). A similar phenotype was seen in A172 cells (data not shown). Changes in morphology, actin dynamics, and cell shape were associated with a decrease in cell motility and invasion of U87 cells (Figure 5B). The inhibition of motility and invasion occurred after 24 hours. In this timeframe effects of STK17A knockdown on proliferation were minimal indicating that the decline in motility and invasion was not due to a decrease in cell proliferation (Figure S5). Interestingly, although STK17A-sh1 is less effective in reducing expression of STK17A as compared to STK17A-sh2, it is more effective at reducing migration and invasion. In contrast, an increase in motility or migration was not detected when STK17A was overexpressed in U87 cells (data not shown). This suggests that U87 cells may already be optimally invasive and motile or that the high basal levels of STK17A in U87 cells is already saturating for motility and invasion. An alternate explanation is that STK17A may be necessary but not sufficient to alter invasion and/or motility.

### STK17A knockdown in GBM decreases clonogenic and anchorage-independent cell growth and increases sensitivity to DNA damaging agents

**Figure 6:**
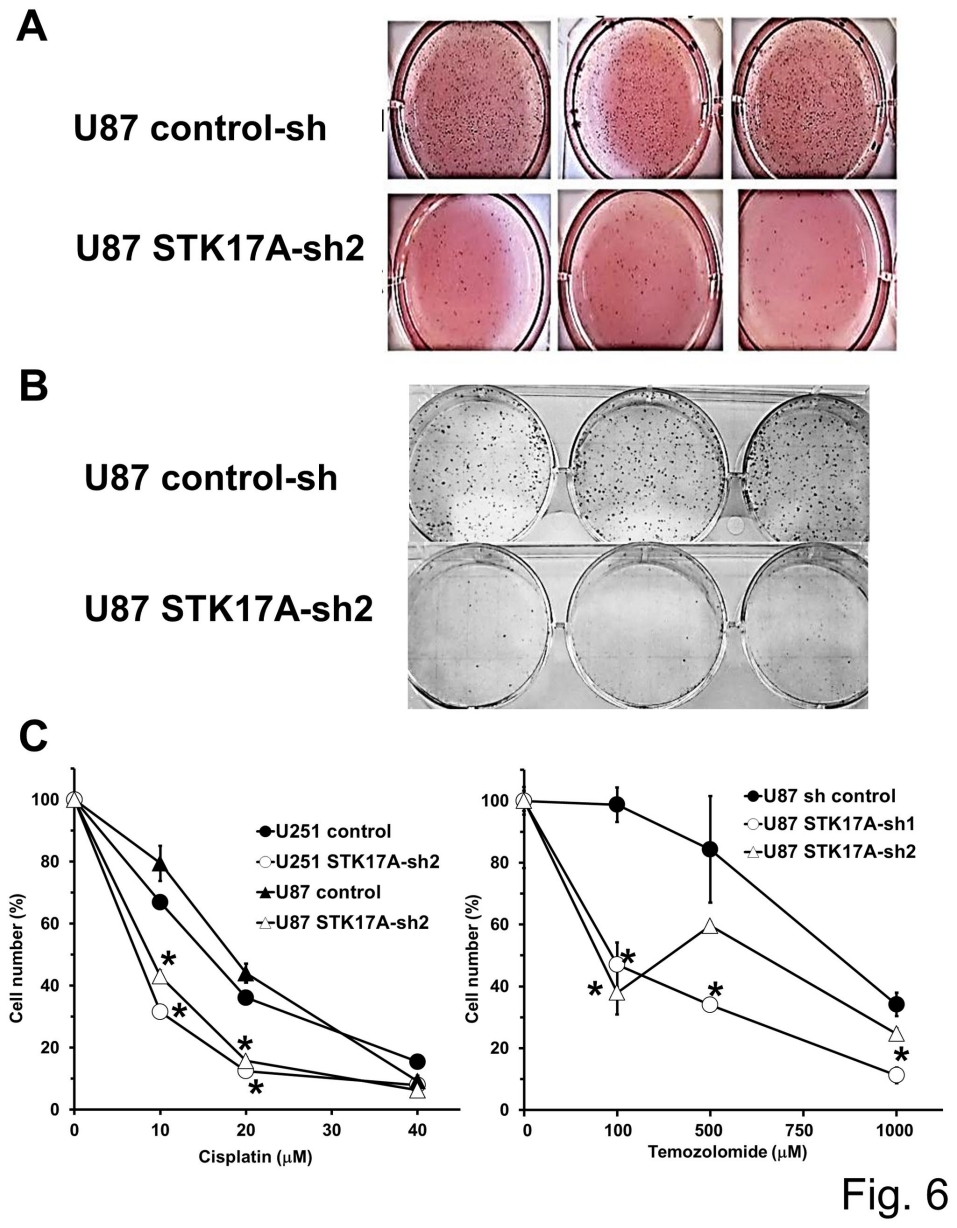
STK17A knockdown decreases oncogenic properties of GBM cells. **A**, STK17A knockdown decreases soft agar colony formation. U87 control or U87 STK17A knockdown cells were suspended in soft agar and cells were stained with MTT reagent after 2 weeks of culture. Representative of two independent experiments. **B**, STK17A knockdown decreases clonogenicity of GBM cells. U87 control or STK17A U87 knockdown cells were plated and stained with Giemsa after 10 days of cell culture. Representative of three independent experiments. **C**, STK17A knockdown sensitizes GBM cells to cisplatin and temozolomide. Left, Dose response after 3 days of cisplatin treatment of U87 or U251 control versus STK17A knockdown cells. Cell proliferation and survival was measured with Cell-Titer Glo reagent. Data points are the average of biological triplicates and error bars are SD. *, p < 0.05. Representative of three independent experiments. Right, Dose response after 3 days of temozolomide treatment of U87 control versus U87 STK17A knockdown cells. Cell proliferation and survival was measured with Cell-Titer Glo reagent. Data points are the average of biological triplicates and error bars are SD. *, p< 0.05. Representative of two independent experiments.

Since STK17A is highly expressed in GBM, the effect of STK17A knockdown on the oncogenic properties of GBM cells was assessed. Knockdown of STK17A caused a substantial reduction in soft agar clone formation and clonogenicity of U87, SF268, A172 and U118 cells (Figure 6, Figure S6 and Figure S7). In addition, knockdown of STK17A sensitized U251 and U87 GBM cells to the DNA damaging agent cisplatin and U87 cells to temozolomide, while overexpression of STK17A in U87 cells resulted in resistance to cisplatin (Figure 6 and Figure S8A). Interestingly, U251 has mutant p53 while U87 has wild-type p53 yet both cell lines are sensitized to cisplatin upon STK17A knockdown. This suggests that the effects of STK17A on sensitivity to cisplatin can occur in the presence or absence of wild-type p53 in GBM cells. This finding is consistent with data generated from a recent systematic analysis of genetic markers of drug resistance in cancer cells which found cisplatin resistance associated with high levels of STK17A expression in a panel of nervous system derived tumor cell lines which included glioma, medulloblastoma and neuroblastoma lines (Figure S8B) [28]. High levels of STK17A expression were also associated with resistance to BID1870 (RSK, PLK1, AURKB inhibitor; 2.0-fold), AZD7762 (CHK1/2 inhibitor, 2.9-fold) and PD0332991 (CDK4/6 inhibitor, 2.1-fold) [28]. These data indicate that STK17A may play a role in promoting tumorigenicity and resistance to chemotherapeutics in GBM cells.

### STK17A expression increases with glioma grade and is associated with decreased patient survival

**Figure 7:**
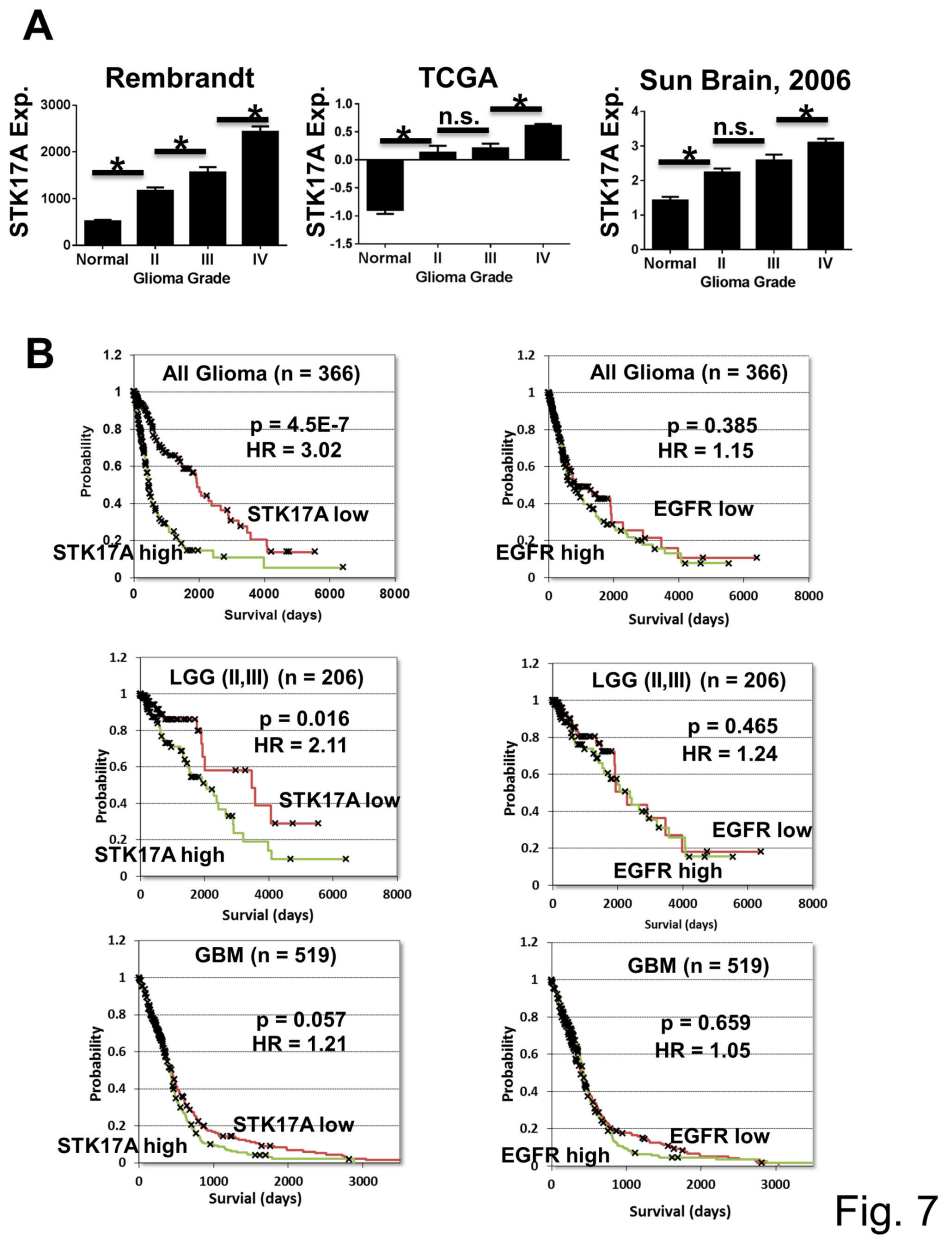
STK17A expression in glioma is associated with high grade and decreased survival. **A**, Expression data was downloaded from the Rembrandt and TCGA databases and data from Sun Brain [19] was downloaded from Oncomine and grouped according to grade. Rembrandt has 21 normal, 99 grade II, 85 grade III and 130 grade IV samples. TCGA has 10 normal, 7 grade II, 20 grade III and 482 grade IV samples and Sun Brain has 26 normal, 45 grade II, 31 grade III and 81 grade IV samples. Error bars are SEM. *, p < 0.02. **B**, STK17A expression is associated with poor overall survival in gliomas. Kaplan-Meier log-rank tests were performed on data obtained from the TCGA database. In all cases high and low expressing groups were divided at the median. All glioma and low grade glioma expression was from RNA-seq data while GBM expression was from Affymetrix microarray data.

In order to assess the clinical relevance of STK17A overexpression in glioma, further analysis was performed using publically available datasets. Three distinct datasets revealed that STK17A expression increases progressively with glioma grade and is highest in the most aggressive and lethal grade IV glioma (GBM) (Figure 7A). Increased expression of STK17A was associated with a highly significant decrease in overall patient survival when assessed by the Kaplan-Meier log-rank test in a combined cohort of all patients with glioma from the TCGA database (Figure 7B). Glioma patients were divided at the median STK17A expression level for this analysis. STK17A resides on chromosome 7 approximately 12 MB from EGFR which is frequently amplified in gliomas [11]. While high STK17A expression is associated with poor survival in glioma, similar analysis did not find an association between high EGFR expression and survival in the same cohort (Figure 7B). Univariant Cox proportional analysis using STK17A and EGFR expression as continuous variables also indicated that high STK17A (Hazard Ratio (HR) = 2.07, p value = 5.48 x 10^-7^), but not high EGFR (HR = 1.07, p value = 0.17), was associated with poor survival (Table A in Table S1). To assess confounding effects associated with STK17A expression, multivariant Cox proportional analysis was performed with age, EGFR level, gender and grade as confounding variables. Since STK17A is expressed in a grade-depended manner (Figure 7A), it was not surprising that grade was a partially confounding variable. However, STK17A expression still independently predicted survival after correcting for grade, albeit with a lower HR and p value (Table B in Table S1). STK17A expression also predicted overall survival after correcting for age, gender and EGFR expression (Table B in Table S1). 

Kaplan-Meier analysis was repeated with a TCGA cohort containing only low grade gliomas (II and III). STK17A expression, but not EGFR expression, again significantly predicted overall patient survival (Figure 7B) and STK17A expression was again shown to be a predictor of survival (HR = 2.29) when corrected for grade, age and EGFR expression upon univariant and multivariant Cox analysis (Table C and Table D in Table S1). STK17A expression did however lose predictive power in a TCGA cohort of only GBM patients which more uniformly express high levels of STK17A. Although Kaplan-Meier analysis still indicated borderline significance of STK17A as a predictor of survival in GBM (Figure 7B) and Cox analysis indicated a significant association between STK17A expression and survival even after correcting for EGFR, gender and molecular phenotype, the HR values were lower as compared to the LGG cohort and significance was lost when correcting for age (Table E and Table F in Table S1). Together the data suggests that STK17A is an independent predictor of survival in glioma patients, but since STK17A expression is highly grade-dependent, grade is likely a partially confounding factor.

Further analysis performed within the low grade glioma cohort indicated that STK17A remains a significant predictor of survival after correcting for the common genetic glioma alterations of IDH1 mutation and PTEN loss (Table G and Table H in Table S1). Further the p53 target gene STK17A remained a predictor of survival after correction for p53 pathway disruption due the p53 mutation, loss of CDKN2A, and amplification of MDM2 and MDM4 (Table G and Table H in Table S1). We were unable to detect any significant trends in STK17A expression associated with p53 pathway alterations except for deletion of CDKN2A (P19ARF). Low grade glioma and high grade GBM tumors with homologous deletion of CDKN2A had significantly higher levels of STK17A, suggesting that those tumors with deactivation of p53 due to CDKN2A loss may compensate by upregulating STK17A (Figure S9). 

## Discussion

GBM is a devastating disease with high lethality despite current treatment modalities [2,5]. Here we have identified STK17A as a novel candidate kinase target in gliomas. Evidence that STK17A promotes glioma progression and survival includes the finding that STK17A is highly overexpressed in a grade-dependent manner in tumor compared to normal brain and other tumor types and that STK17A knockdown decreases cell proliferation, soft agar colony formation and clonogenicity and sensitizes GBM cells to genotoxic stress. Further, high levels of STK17A were associated with poor survival in patients with glioma indicating that STK17A may have clinical relevance as a therapeutic target in these neoplasms. 

We previously showed that STK17A is a p53 target gene and is induced in a p53-dependent manner in several cell contexts including testicular cancer cells [18]. Our current and previous work suggests that STK17A may have context dependent activities since STK17A was associated with cisplatin sensitivity in testicular cancer cells which express very low levels of basal STK17A that is highly induced upon genotoxic stress [18]. In contract STK17A is highly basally expressed in GBM cell lines, even in U251, U118 and U563 cells mutant for p53, and confers chemotherapy resistance. This difference may relate to the different levels of STK17A or the widely distinct cell contexts of testicular germ cell cancer compared to GBM. Another possibility relates to potential activities of STK17A that are known to be context-dependent. For example, other kinases in the DAP-family are known to regulate autophagy which can be pro- or anti-apoptotic depending on cell context and level of stress [29,30]. It is noteworthy that p53 itself can mediate either chemotherapy resistance or chemotherapy sensitivity depending on the cancer cell context and level of stress [31,32]. 

It should be noted that our analysis found an association between STK17A expression and survival using an unbiased means of defining high and low STK17A expression as either above or below the median expression value in each data set (Figure 7B) or treating STK17A expression as a continuous variable (Tables A-H in Table S1). Defining high and low EGFR expression above and below its median expression value failed to show an association of high EGFR with poor survival in all glioma, low grade glioma, and GBM. Treating EGFR expression as a continuous variable only showed a significant association with poor survival in the low grade glioma dataset (Tables A-H in Table S1). However bifurcating patients into two groups, those that either have an EGFR amplification, activating mutation or overexpression above 2-fold versus those that do not results in a significant association of the EGFR gain of function group with poor survival. For low grade glioma the association has a HR of 2.0, p value = 0.041 and for GBM the association has a HR of 1.37, p = 0.0033. This result is consistent with the known association of EGFR gain of function with poor survival in GBM [11]. 

In addition to the association of high STK17A expression with poor patient survival, we have shown in U87 cells that STK17A knockdown decreases cell invasion and motility and alters cell shape and actin dynamics. While several molecular functions of the DAP-family have been proposed, the most studied is the interaction with the cytoskeleton through phosphorylation of the regulatory subunit of myosin light chain (MLC), leading to modulation of actin stress fibers, adhesion junctions and cell motility [12,15,17,33]. Extending these findings to STK17A must be taken with caution since STK17A lacks the cytoskeletal-related regulatory and targeting domains of DAPK1. However, it is known that STK17A can phosphorylate MLC *in vitro* [14]. In addition Drosophila has only a single DAP kinase like gene called DRAK which is most closely related to STK17A [27]. Recent genetic evidence suggests that DRAK is involved in regulating epithelial tissue morphology, cell shape and adhesion, organ growth and cytoskeletal dynamics through phosphorylation of the Drosophila ortholog of MLC (sqh) [25-27]. Further detailed study will be required to uncover the exact mechanism(s) by which STK17A regulates cytoskeletal dynamics and cell motility and to uncover the extent by which these functions impact the cell proliferation, survival and tumorigenic promoting properties of STK17A in GBM cells. 

In conclusion, this study indicates that STK17A is overexpressed in gliomas in a manner that corresponds with increasing tumor grade and malignancy. STK17A also promotes cell proliferation, survival and resistance to chemotherapy in GBM cells and STK17A has effects on cell shape, actin dynamics, invasion and motility. The association of STK17A expression with patient survival suggests that STK17A is a potentially important new kinase target in glioma. In summary, we have uncovered an unrecognized role for STK17A in glioma biology. Targeting STK17A may lead to the development of new therapies for GBM and other cancers and may provide a new strategy to sensitize cancers to existing therapies. 

## Materials and Methods

### Cell proliferation, soft agar and clonogenicity assays

Cells were cultured in DMEM (Gibco) with 10% FBS. Human glioblastoma cell lines U87, SNB19, SNB44, SNB75, U118, U563, A172 and SNB19 were purchased from ATCC. Human glioblastoma cell line SKMG3 was obtained from Memorial Sloan Kettering Cancer Center and human glioblastoma cell lines SF268 and SF767 were from the Brain Tumor Research Center, University of California San Francisco. Human osteosarcoma (U2OS), embryonal carcinoma (NT2/D1), breast cancer (MCF7) and colon cancer (HCT116) were purchased from ATCC. Human non-small cell cancer cell lines Hop62, A549 and H23 were obtained from the Developmental Therapeutics Program at the National Cancer Institute. Cell proliferation and survival was assessed by manually counting trypan blue viable cells with a hemocytometer. Plating errors and differences in cell adherence were controlled for by normalizing the number of viable cells counted at 4.5 days after plating with the number of viable cells counted 0.5 days after plating. Cell proliferation and survival after drug treatment was assessed with Cell-Titre Glo (Promega). Cisplatin and temozolomide were purchased from Sigma. Soft agar assays were performed as previously described [34]. Briefly, cells were plated in DMEM containing 10% FBS and 0.26% Seakem ME Agarose (Cambrex Bioscience). A total of 1.5 × 10^4^ viable, trypan blue-excluding cells were plated per well of a six well plate. A lower layer of 1% agarose in DMEM and 10% FBS was used. Cells were cultured every 4 days with fresh media. After 2 weeks of culture, colonies were stained with a 5-mg/ml solution of MTT Reagent (Sigma) in PBS. For clonogenic assays 1.0 × 10^3^ viable, trypan blue-excluding cells were plated per well of a six well plate. Cells were cultured every 4 days with fresh media and colonies were fixed in methanol and stained with Giemsa stain after 10 days. 

### RNAi knockdown

Lentiviral silencing shRNAs targeting human STK17A (TRCN0976, STK17A-sh1 and TRCN0975, STK17A-sh2) were purchased from Open Biosystems along with pLKO.1 control. The sequence of STK17Ash1 is AGGCTCTTGAATACTGCTCTG and the sequence for STK17A-sh2 is TAGTTCAAGTACAGCAATCTC. Lentiviral stocks were generated from 293T cells as described previously [35]. GBM cells were cultured with lentiviral stocks for 24 h and stable pools were selected with 1.0 µg/ml puromycin. 

### Western analysis and real-time PCR

SYBR green-based real-time PCR (Applied Biosystems) was employed using the ddCT method normalized to GAPDH. STK17A primers were 5’-GCTGGGGAGAGCGGGTGTTT-3’ and 5’-CTGGCCGCCTGTTCACTCCG-3’. GAPDH primers were 5’-GATTCCACCCATGGCAAATT-3’ and 5’-GATGGTGATGGGATTTCCATTG-3’. For Western analysis cells were lysed in radioimmune precipitation buffer and separated by SDS-PAGE. Antibodies to actin (sc01615, Santa Cruz), GAPDH (sc47724, Santa Cruz) and STK17A (IMG-157-1, Imgenex) were used. Despite the effects of STK17A on cell shape, similar results were obtained when actin and GAPDH were used to controls for loading (Figure S2). Archival frozen GBM samples were procured through the pathology core facility of the Norris Cotton Cancer Center. These samples were primary tumor tissue sections obtained at the time of initial surgery and prior to therapy from patients subsequently diagnosed with type IV GBM. Samples were processed for RNA extraction by freezing in liquid nitrogen and pulverizing with a mortar and pestle before dissolving in Tri-Reagent (Sigma). Normal RNA was purchased from Clontech. NB26 refers to human fetal brain total RNA (catalog #636526). NB30 refers to human brain total RNA (catalog #636530). 

### Migration, invasion assays and actin staining

Invasion (200,000 cells) and migration (50,000 cells) assays were performed using 6-well 8.0 µM trans-well inserts (BD Biosciences). For the invasion assay, the inserts were coated with growth factor reduced matrigel (diluted 1 to 1 with serum free DMEM). The upper part of the insert was exposed to 1% FBS and the lower part to 10% FBS. After 24 hours, the upper part of the insert was cleaned using cotton swabs and the insert was cut and mounted using DAP1 containing florescent mounting solution (Vector Biolabs). Cells were counted in ten random fields using an epiflourescent microscope on 20X magnification. For the migration assay, the assay was performed exactly as described above with the exception of matrigel addition. For actin staining cells were fixed with 4% paraformaldehyde for 20 minutes and permeabilized with 0.25% Triton-X-100 for 20 minutes. Cells were stained with 50 nM tetramethylrhodamine isothiocyanate (TRITC)-phalloidin (Sigma) for 1 hour. Cell nuclei were then stained with DAPI mounting solution. Cells were also plated at 50,000 and 200,000 per 6-well plate and counted with trypan blue 24 hours later to control for proliferation. 

### *In silco* and Survival analysis

STK17A expression from Liang et al., French et al., Murat et al., and Bredel et al. [20-23] was obtained from Oncomine (https://www.oncomine.com). STK17A expression in 1062 cancer cell lines was obtained through the Cancer Cell Line Encyclopedia (http://www.broadinstitute.org/ccle/home). For expression analysis according to grade, Rembrandt Affymetrix expression data was downloaded from the CA-integrator website (https://caintegrator.nci.nih.gov/caintegrator/) and normalized Agilent expression data for normal brain, glioblastoma multiforme (GBM) and brain low grade gliomas (LGG) was downloaded from the TCGA website (https://tcga-data.nci.nih.gov/tcga/tcgaHome2.jsp). Clinical and gene expression data for survival analysis was downloaded from the TCGA website on April 30, 2013. Kaplan-Meier log-rank tests were performed using WinSTAT and patients were divided into low or high expression groups at the median expression value in all cases. RNA-seq expression data for (GBM) and (LLG) was combined for Kaplan-Meier analysis of all gliomas. Analysis for LGG (grade II and III) used RNA-seq data. STK17A expression for Kaplan-Meier analysis of GBM was in the form of normalized Affymetrix microarray data. Univariant and multivariant Cox proportional analysis was performed using the above datasets in WinSTAT. Multivariant analysis utilized the direct method. STK17A and EGFR expression were treated as continuous variables of the log 2 transformed RNA-seq or normalized Affymetrix data for Cox analysis. Age was treated as a continuous variable. Information on mutations and the molecular phenotype of tumors was downloaded from the cBioPortal website (http://www.cbioportal.org/public-portal/) which necessitated decreasing the number of patients for Cox regression since this data was only available for a subset of LGG and GBM tumors. 

### Ethics Statement

Use of archival GBM samples was approved by the Committee for the Protection of Human Subjects at Dartmouth-Hitchcock Medical Center (CPHS #23445). Approval for use of samples for research purposes was obtained by written consent.

### Statistics

When a value for statistical significance is provided, a two-sample, two-tailed *t* test assuming unequal variance was performed.

## Supporting Information

Figure S1 **STK17A is overexpressed in glioblastoma and anaplastic oligodendroglioma compared to normal brain.** **A**, Relative STK17A expression from Liang et al., French et al., Murat et al., and Bredel et al. [20-23] demonstrates increased STK17A expression in GBM and oligodendroglioma (AO) compared to normal brain. Data was obtained through the Oncomine database. **B**, STK17A is highly expressed in glioma cell lines compared to other cancer types. Data was obtained through the Cancer Cell Line Encyclopedia (CCLE).
(TIF)

Figure S2 **STK17A protein is overexpressed in GBM cell lines compared to NT2/D1, U2OS, H23, Hop62 and MCF7 cells.** **A**, **B**, Western analysis comparing STK17A expression in U87, SNB44, SNB75, SKMG3, U118, U251 and U563 GBM cells compared to NT2/D1 human embryonal carcinoma, U2OS osteosarcoma, H23 and Hop62 lung cancer and MCF7 breast cancer cells. Arrow indicates STK17A specific band identified by the diminished signal in U87 cells stably expressing STK17A shRNA.
(TIF)

Figure S3 **STK17A knockdown results in decreased GBM cell proliferation.** U563 and A172 GBM cells stably expressing STK17A shRNA or a control shRNA were assessed for changes in cell proliferation by cell counting 4.5 days after plating. Cell counts were normalized to cell counts after 0.5 days of plating to control for plating errors and differences in cell adherence. Bars are the average of three biological replicates and error bars are SEM. *, p < 0.01. Note A172 cells with STK17A knockdown had a borderline significant decrease in proliferation; p = 0.072. To the right of each graph the extent of STK17A knockdown was assessed by Western analysis.
(TIF)

Figure S4 **STK17A knockdown or overexpression alters the morphology of A172 GBM cells.** **A**, Representative micrographs of parental A172 cells with control shRNA, A172 cells with STK17A shRNA, and A172 cells stably overexpressing STK17A. Pictures were taken at 10X magnification on a NIKON ELWD microscope. **B**, Western analysis documenting STK17A knockdown and overexpression in engineered A172 cells.
(TIF)

Figure S5 **STK17A knockdown does not affect U87 cell proliferation at 24 hours.** Cells were plated at a density of 50,000 (**A**) and 200,000 (**B**) per 6-well plate to match conditions of migration and invasion assays, respectively. Trypan blue excluding cells were manually counted 24 hours later.
(TIF)

Figure S6 **STK17A knockdown leads to decreased soft agar colony formation in SF268 GBM cells.** SF268 control or SF268 STK17A knockdown cells were suspended in soft agar and cells were stained with the MTT assay after 2 weeks of culture. STK17A knockdown in SF268sh1 cells is depicted in Figure 3 of the main text.
(TIF)

Figure S7 **Effects of STK17A knockdown on clonogenicity of GBM cells.** Stable control shRNA or STK17A-shRNA cells were plated and stained with Giemsa stain after 10 days of cell culture. **A**, SF268; **B**, A172; **C**, U118. Western analysis documenting STK17A knockdown is depicted in Figure S3 and in Figure 3 of the main text.
(TIF)

Figure S8 **STK17A overexpression is associated with resistance to cisplatin.** **A**, U87 control cells or U87 cells stably overexpressing STK17A were treated with indicated doses of cisplatin for three days and then cell proliferation and survival was measured with the Cell-Titer Glo assay. Data points are the average of biological triplicates and error bars are SD. **B**, Data obtained through the Oncomine database from Garnett et al. [28], demonstrating high levels of STK17A mRNA are associated with cisplatin resistance in CNS cell lines.
(TIF)

Figure S9 **CDKN2A loss is associated with high STK17A expression in glioma.** TCGA expression data downloaded from the TCGA database was grouped according to CDKN2A status. Loss is defined as homologous deletion or mutation. **A**, Low grade glioma is expression data in the form of log 2 transformed normalized RNA-seq levels. CDKN2A wild-type represents 18 samples and CDKN2A loss represents 147 samples. **B**, Glioblastoma is expression data in the form of normalized Affymetrix signal. CDKN2A wild-type represents 303 samples and CDKN2A loss represents 225 samples. *, p < 0.001.
(TIF)

Table S1 **Combined files tables (**A**-**H**) of Cox proportional analyses for all glioma, low grade glioma and glioblastoma.** Table A, Univariant Cox proportional analysis for all glioma overall survival (TCGA). Table B, Multivariant Cox proportional analysis for all glioma overall survival (TCGA). Table C, Univariant Cox proportional analysis for low grade glioma overall survival (TCGA). Table D, Multivariant Cox proportional analysis for low grade glioma overall survival (TCGA). Table E, Univariant Cox proportional analysis for glioblastoma overall survival (TCGA). Table F, Multivariant Cox proportional analysis for glioblastoma overall survival (TCGA). Table G, Univariant Cox proportional analysis with genetic alterations for low grade glioma overall survival (TCGA). Table H, Multivariant Cox proportional analysis with genetic alterations for low grade glioma overall survival (TCGA).
(XLSX)
