# Supplementary figures and images for: Serine/Threonine Kinase 17A Is a Novel Candidate for Therapeutic Targeting in Glioblastoma
Source: PLoS One. 2013 Nov 28;8(11):e81803. doi: 10.1371/journal.pone.0081803 (PMC3842963; doi:10.1371/journal.pone.0081803)

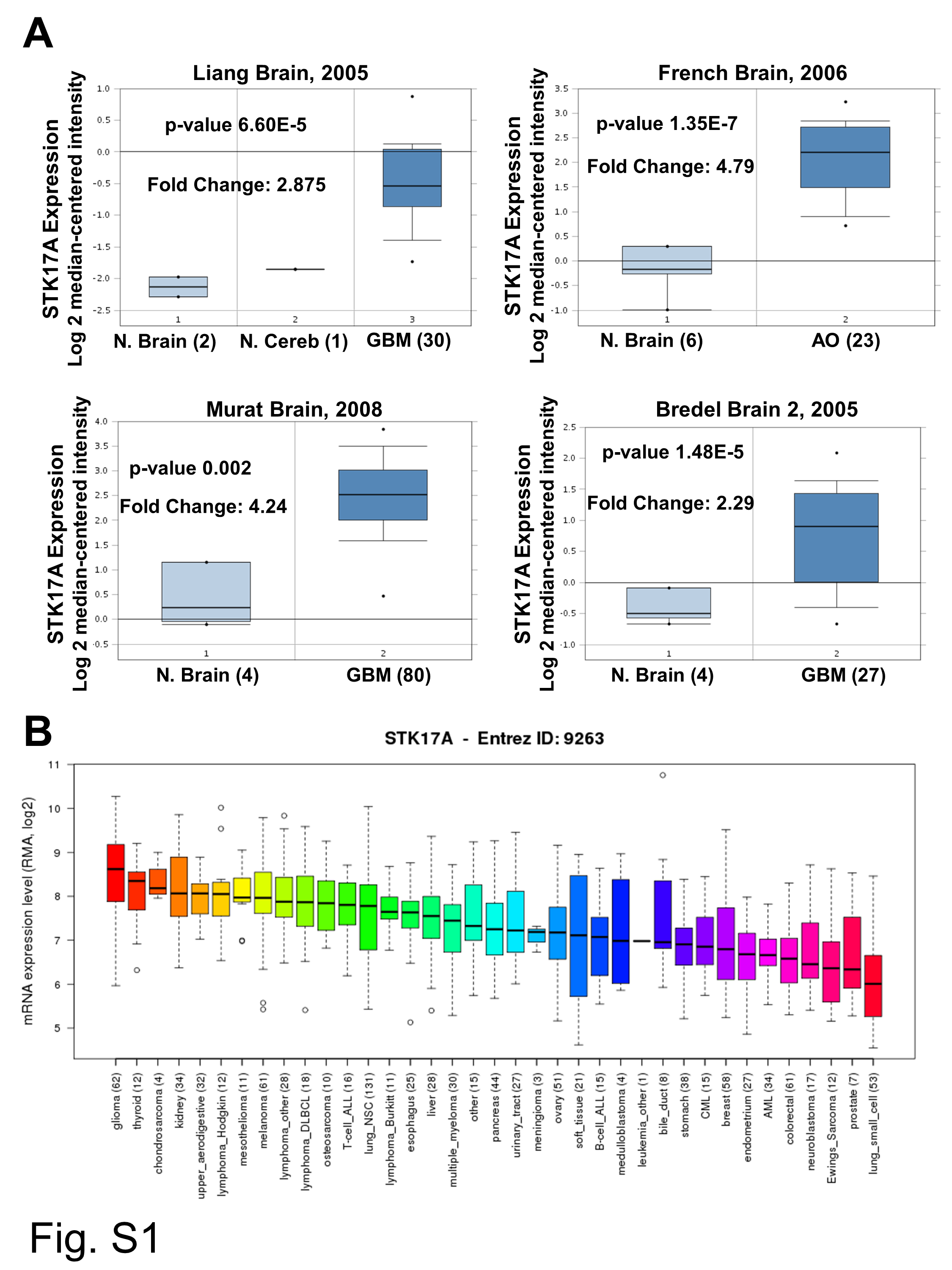

Supplement: Figure S1 — STK17A is overexpressed in glioblastoma and anaplastic oligodendroglioma compared to normal brain. A, Relative STK17A expression from Liang et al., French et al., Murat et al., and Bredel et al. [20-23] demonstrates increased STK17A expression in GBM and oligodendroglioma (AO) compared to normal brain. Data was obtained through the Oncomine database. B, STK17A is highly expressed in glioma cell lines compared to other cancer types. Data was obtained through the Cancer Cell Line Encyclopedia (CCLE). (TIF) [file pone.0081803.s001.tif]

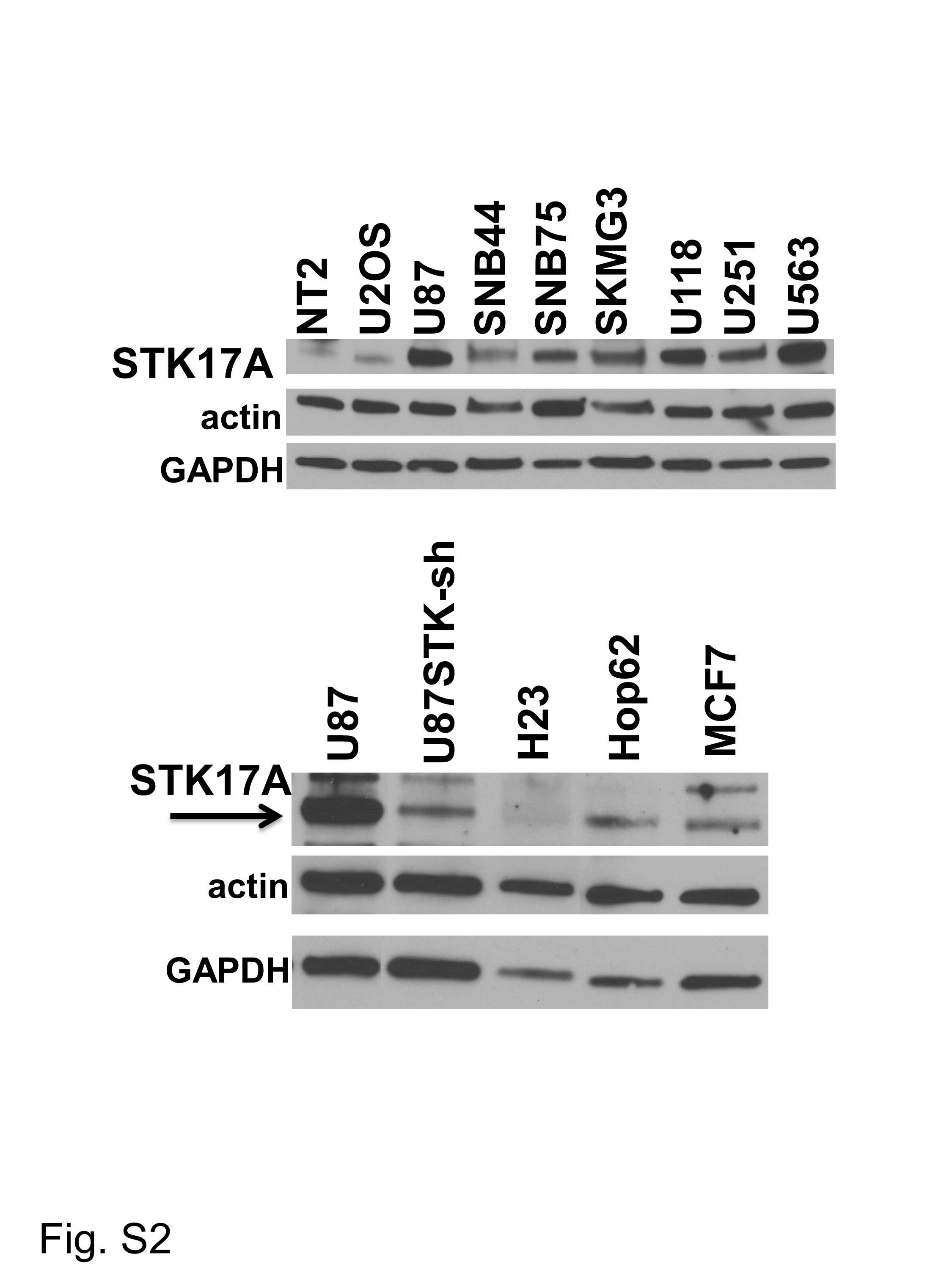

Supplement: Figure S2 — STK17A protein is overexpressed in GBM cell lines compared to NT2/D1, U2OS, H23, Hop62 and MCF7 cells. A, B, Western analysis comparing STK17A expression in U87, SNB44, SNB75, SKMG3, U118, U251 and U563 GBM cells compared to NT2/D1 human embryonal carcinoma, U2OS osteosarcoma, H23 and Hop62 lung cancer and MCF7 breast cancer cells. Arrow indicates STK17A specific band identified by the diminished signal in U87 cells stably expressing STK17A shRNA. (TIF) [file pone.0081803.s002.tif]

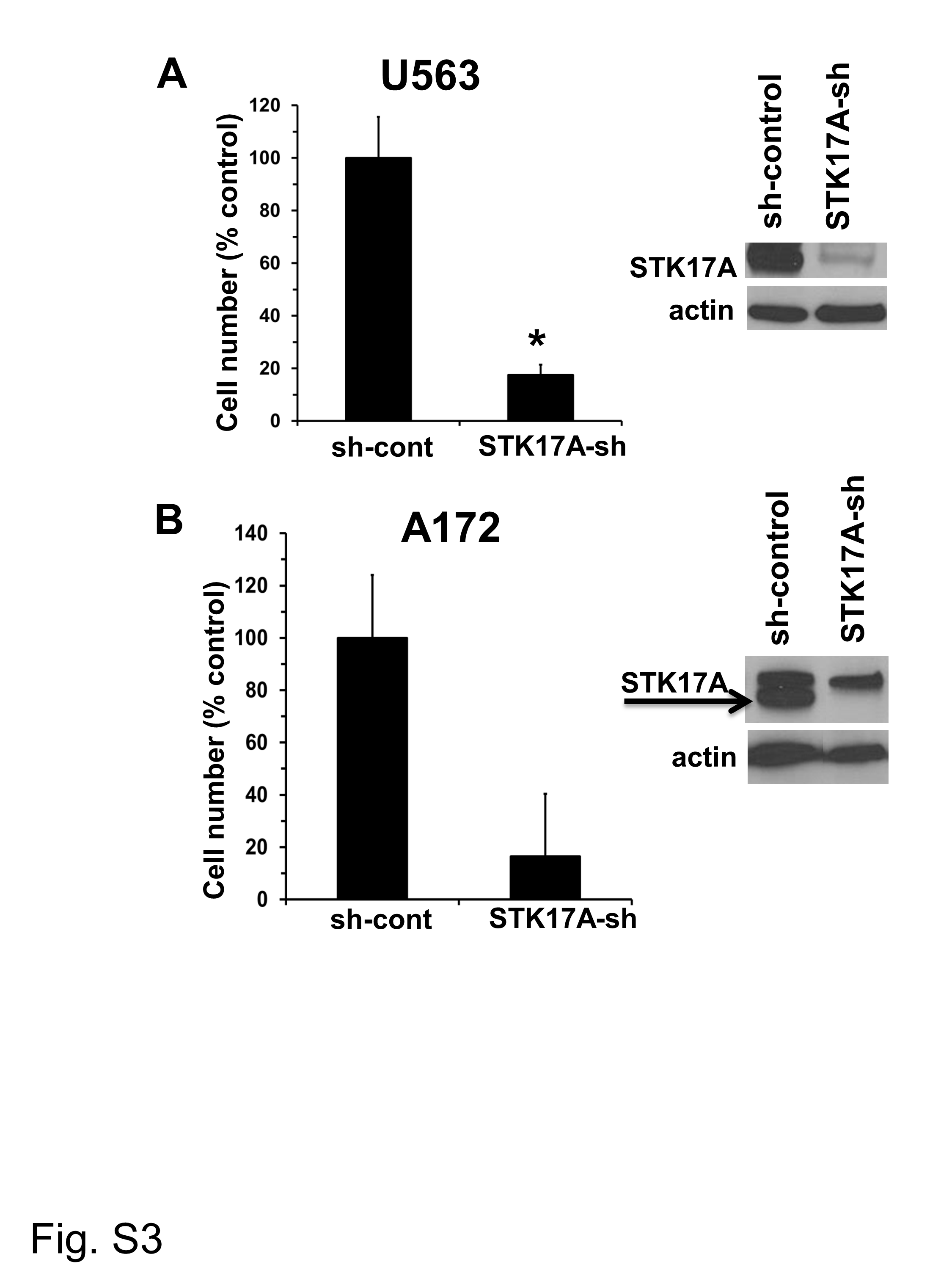

Supplement: Figure S3 — STK17A knockdown results in decreased GBM cell proliferation. U563 and A172 GBM cells stably expressing STK17A shRNA or a control shRNA were assessed for changes in cell proliferation by cell counting 4.5 days after plating. Cell counts were normalized to cell counts after 0.5 days of plating to control for plating errors and differences in cell adherence. Bars are the average of three biological replicates and error bars are SEM. *, p < 0.01. Note A172 cells with STK17A knockdown had a borderline significant decrease in proliferation; p = 0.072. To the right of each graph the extent of STK17A knockdown was assessed by Western analysis. (TIF) [file pone.0081803.s003.tif]

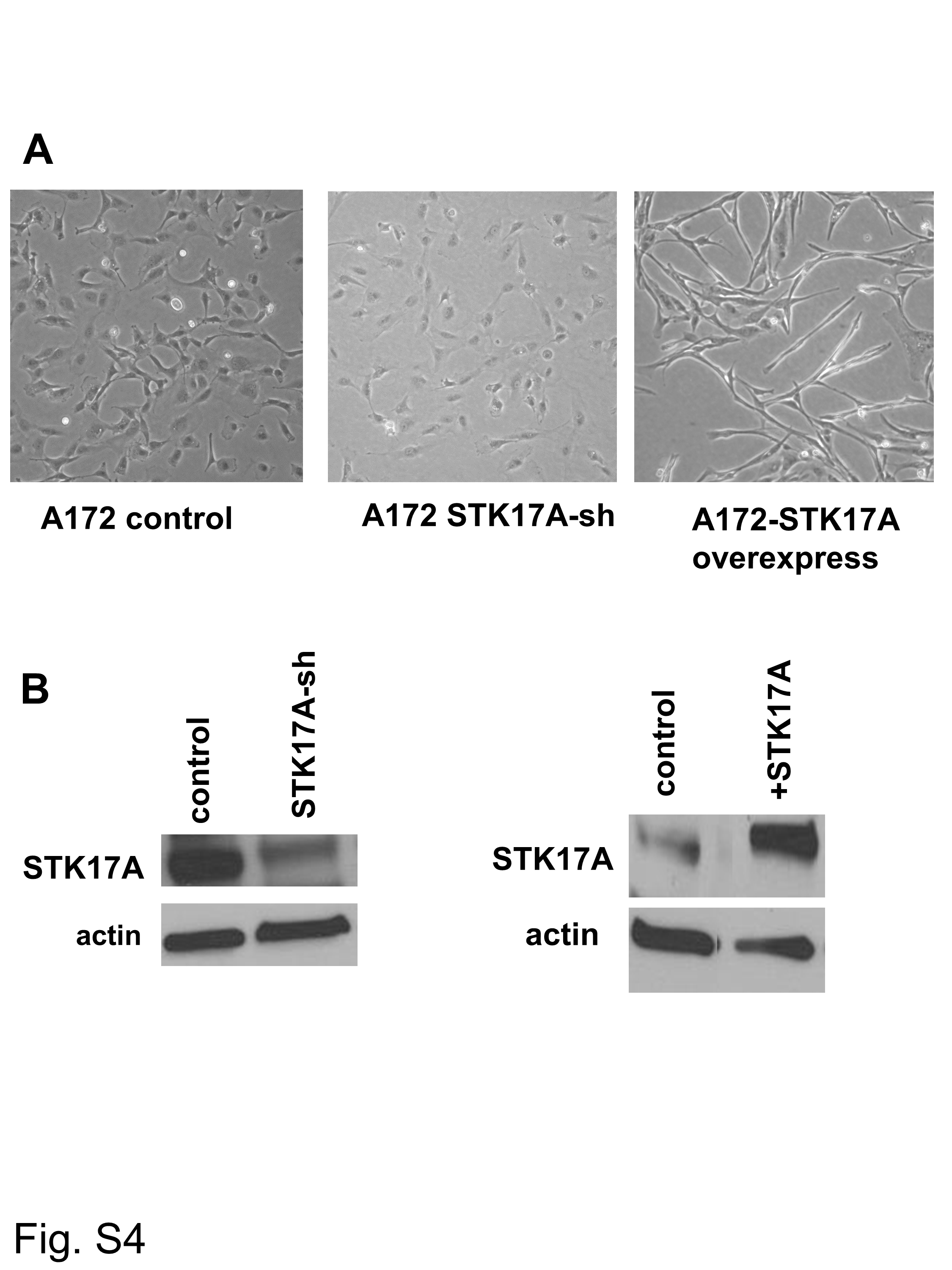

Supplement: Figure S4 — STK17A knockdown or overexpression alters the morphology of A172 GBM cells. A, Representative micrographs of parental A172 cells with control shRNA, A172 cells with STK17A shRNA, and A172 cells stably overexpressing STK17A. Pictures were taken at 10X magnification on a NIKON ELWD microscope. B, Western analysis documenting STK17A knockdown and overexpression in engineered A172 cells. (TIF) [file pone.0081803.s004.tif]

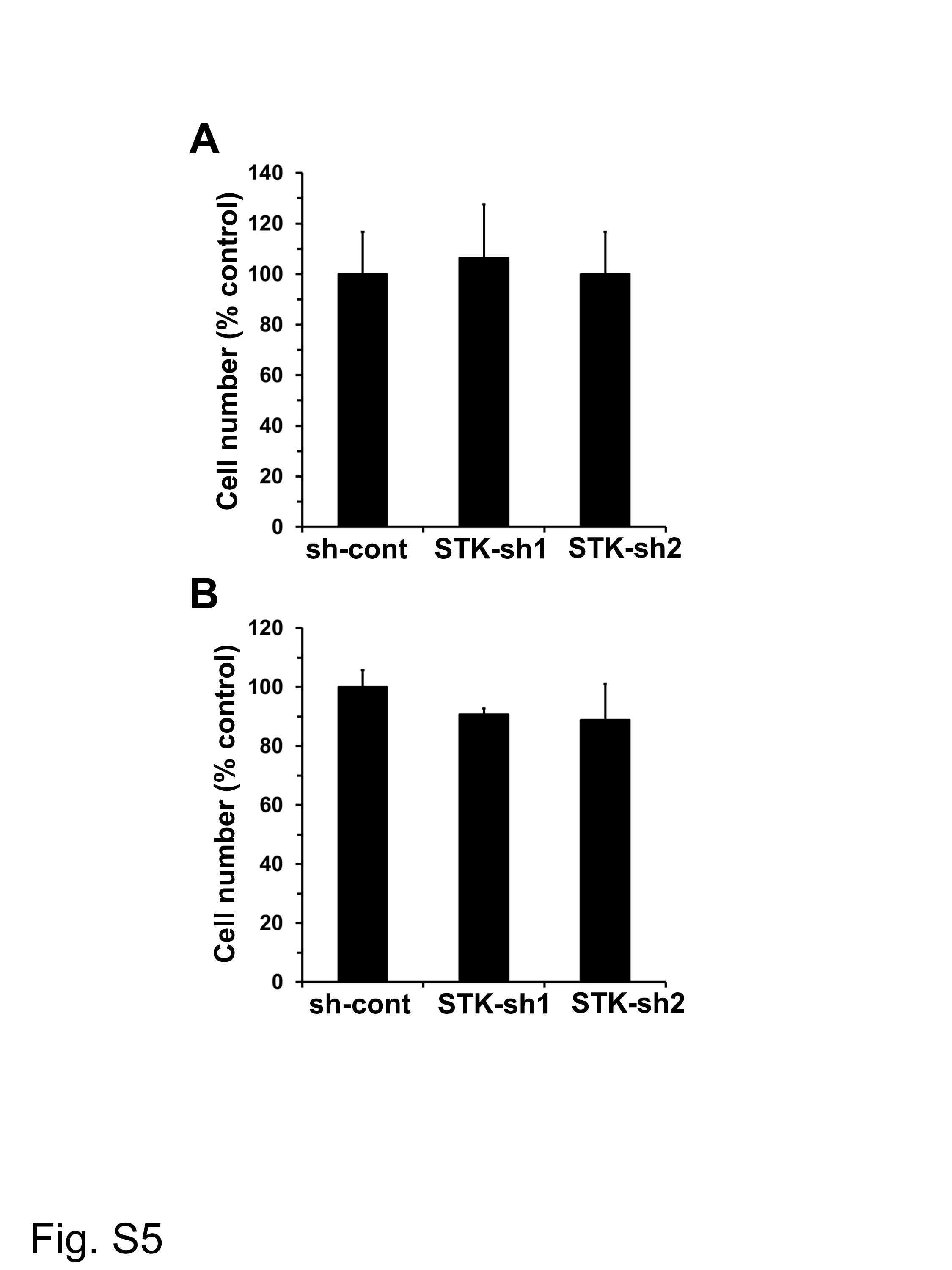

Supplement: Figure S5 — STK17A knockdown does not affect U87 cell proliferation at 24 hours. Cells were plated at a density of 50,000 (A) and 200,000 (B) per 6-well plate to match conditions of migration and invasion assays, respectively. Trypan blue excluding cells were manually counted 24 hours later. (TIF) [file pone.0081803.s005.tif]

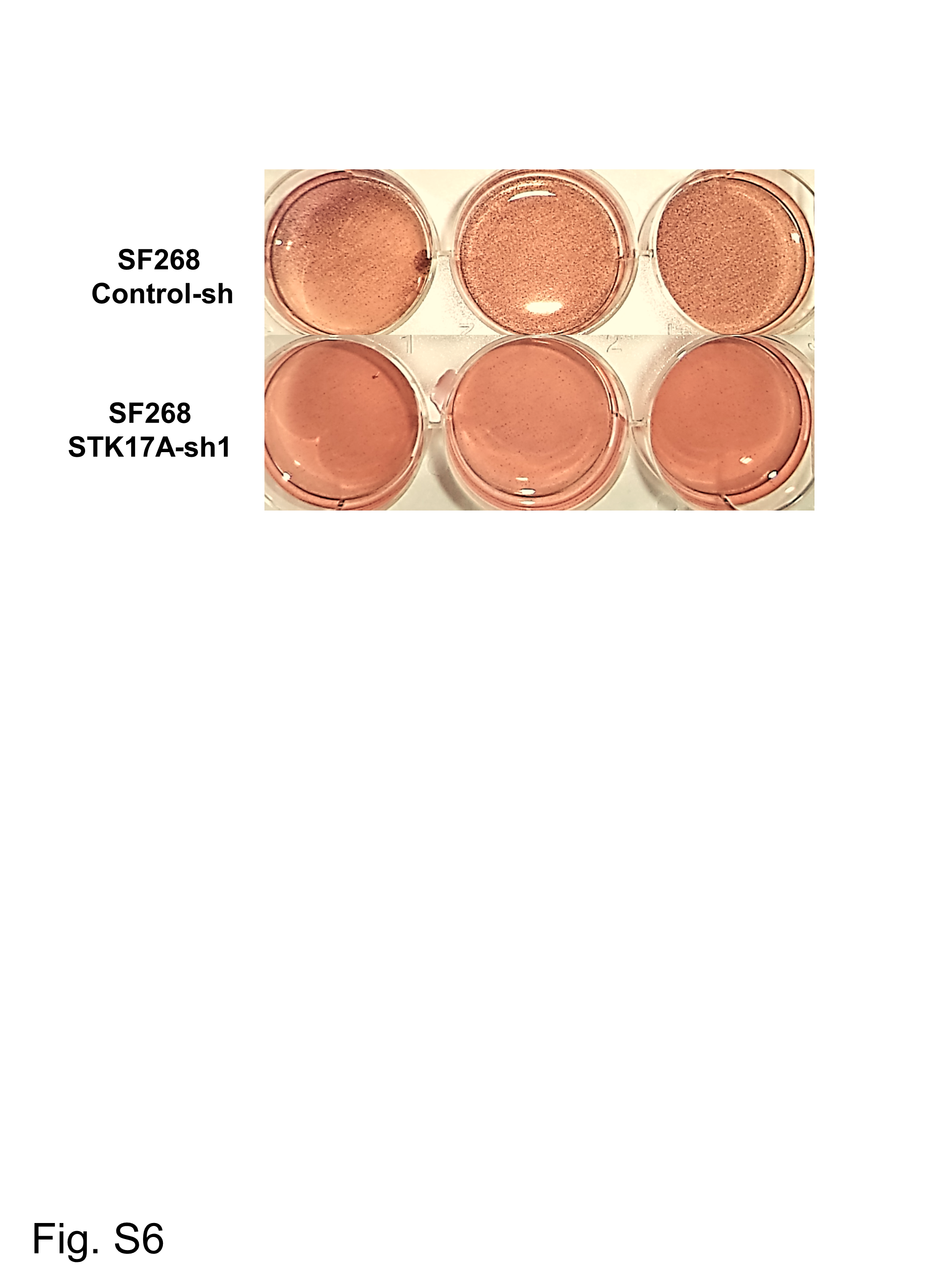

Supplement: Figure S6 — STK17A knockdown leads to decreased soft agar colony formation in SF268 GBM cells. SF268 control or SF268 STK17A knockdown cells were suspended in soft agar and cells were stained with the MTT assay after 2 weeks of culture. STK17A knockdown in SF268sh1 cells is depicted in Figure 3 of the main text. (TIF) [file pone.0081803.s006.tif]

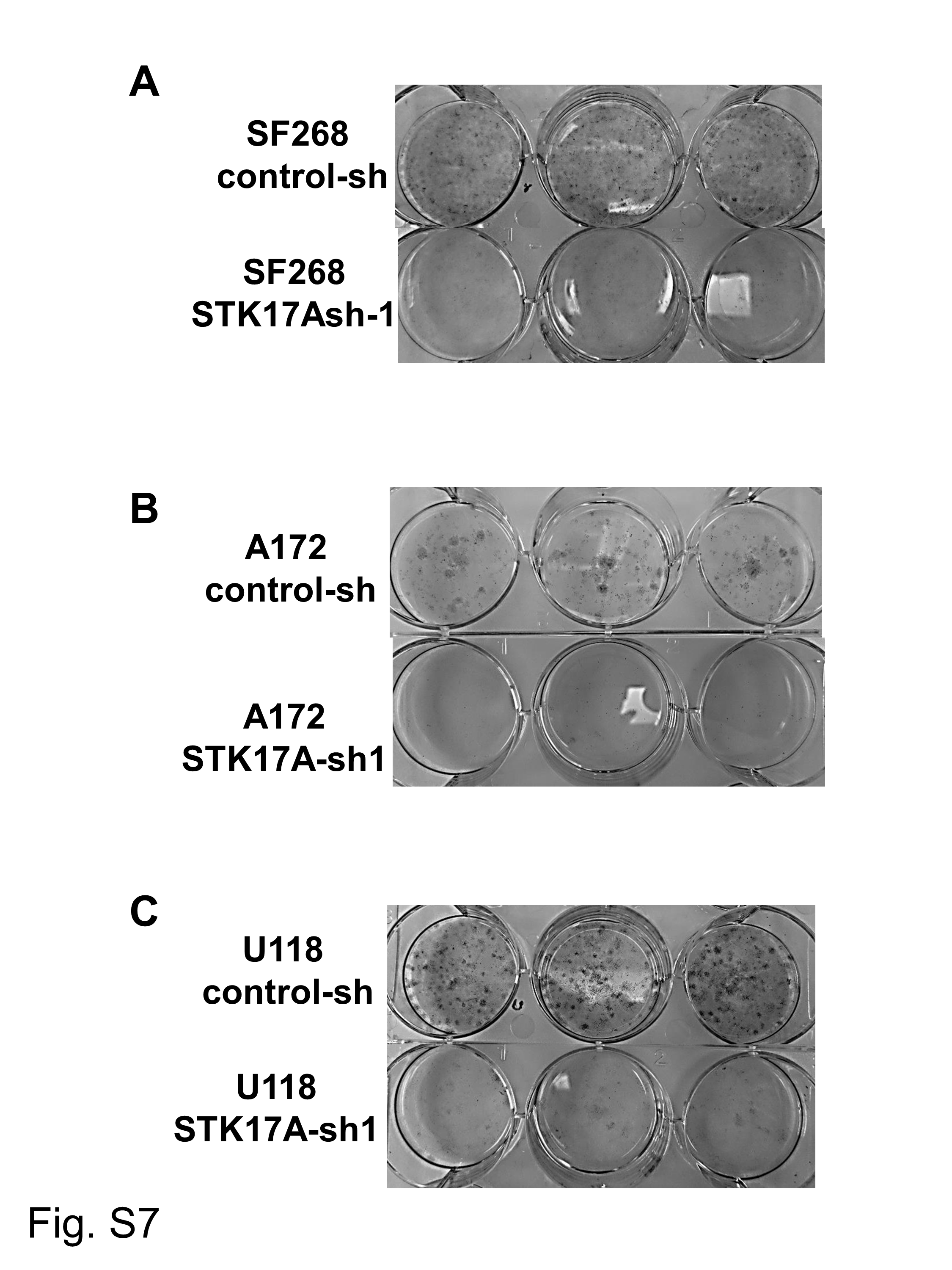

Supplement: Figure S7 — Effects of STK17A knockdown on clonogenicity of GBM cells. Stable control shRNA or STK17A-shRNA cells were plated and stained with Giemsa stain after 10 days of cell culture. A, SF268; B, A172; C, U118. Western analysis documenting STK17A knockdown is depicted in Figure S3 and in Figure 3 of the main text. (TIF) [file pone.0081803.s007.tif]

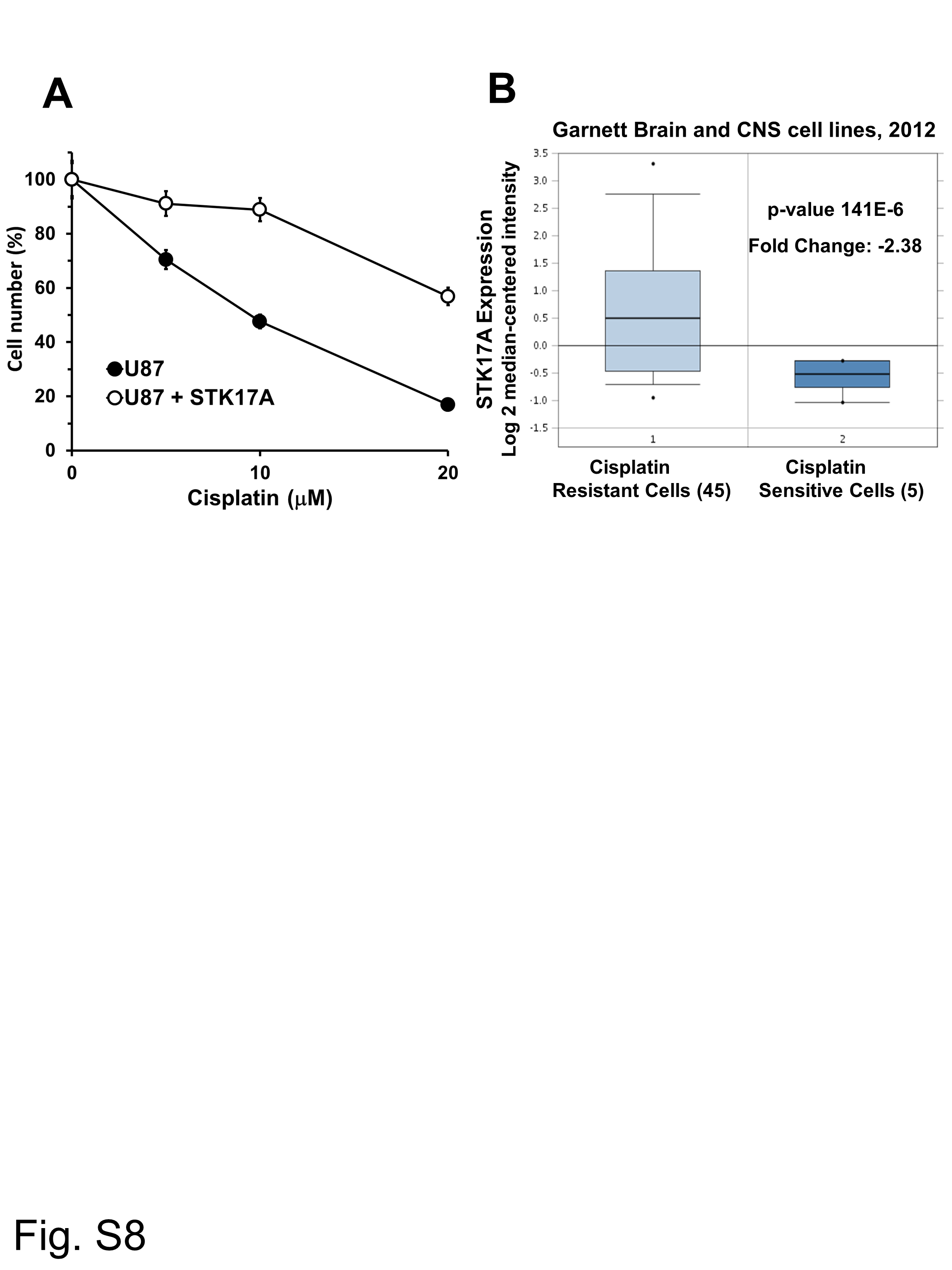

Supplement: Figure S8 — STK17A overexpression is associated with resistance to cisplatin. A, U87 control cells or U87 cells stably overexpressing STK17A were treated with indicated doses of cisplatin for three days and then cell proliferation and survival was measured with the Cell-Titer Glo assay. Data points are the average of biological triplicates and error bars are SD. B, Data obtained through the Oncomine database from Garnett et al. [28], demonstrating high levels of STK17A mRNA are associated with cisplatin resistance in CNS cell lines. (TIF) [file pone.0081803.s008.tif]

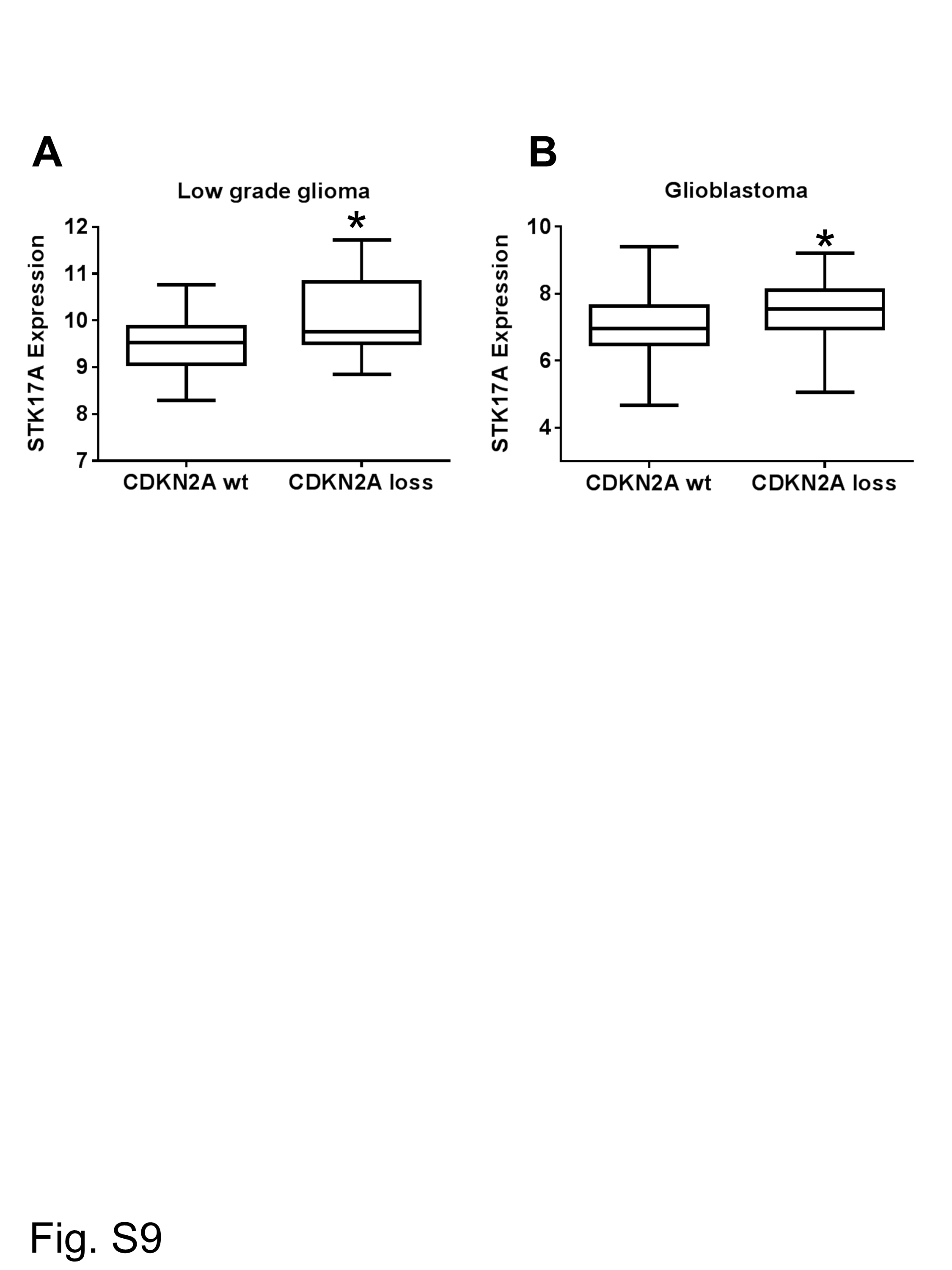

Supplement: Figure S9 — CDKN2A loss is associated with high STK17A expression in glioma. TCGA expression data downloaded from the TCGA database was grouped according to CDKN2A status. Loss is defined as homologous deletion or mutation. A, Low grade glioma is expression data in the form of log 2 transformed normalized RNA-seq levels. CDKN2A wild-type represents 18 samples and CDKN2A loss represents 147 samples. B, Glioblastoma is expression data in the form of normalized Affymetrix signal. CDKN2A wild-type represents 303 samples and CDKN2A loss represents 225 samples. *, p < 0.001. (TIF) [file pone.0081803.s009.tif]
